# Commentary: Effects of occlusion pressure on hemodynamic responses recorded by near-infrared spectroscopy across two visits

**DOI:** 10.3389/fphys.2024.1504497

**Published:** 2024-11-01

**Authors:** Nicholas Rolnick, Jim McEwen, Victor De Queiros

**Affiliations:** ^1^ The Human Performance Mechanic, New York, NY, United States; ^2^ CUNY Lehman College, Bronx, NY, United States; ^3^ The BFR PROS, New York, NY, United States; ^4^ School of Biomedical Engineering and Departments of Orthopedics and Electrical and Computer Engineering, University of British Columbia, Vancouver, BC, Canada; ^5^ Graduate Program in Health Sciences, Federal University of Rio Grande do Norte (UFRN), Natal, Brazil

**Keywords:** BFR training, commentary, arterial occlusion pressure, limb occlusion pressure, reporting

## Introduction

Ischemic preconditioning (IPC) involves brief cycles of ischemia and reperfusion that can be applied prior to physical testing to either enhance physical performance or reduce exercise-induced muscle damage (Franz et al., 2018; Salvador et al., 2016). Ischemia is typically induced using inflatable cuffs placed on the proximal regions of the upper or lower limbs. Many studies use arbitrary pressures (e.g., 220 mmHg) (Salvador et al., 2016); however, this method poses challenges since the same absolute pressure can lead to varying levels of tissue pressure. Factors like limb circumference and cuff size can significantly influence the pressure required to achieve arterial occlusion (de Queiros et al., 2024). As a result, some researchers, such as Desanlis et al. (2024), have focused on refining the standardization of cuff pressures in IPC interventions (Desanlis et al., 2024). Their study aimed to explore how different occlusion pressures affect hemodynamic responses, contributing to more accurate pressure prescription during IPC. While we commend the authors for their valuable efforts, there are some important considerations to keep in mind when interpreting the findings.

## Methodological considerations about the study

The study evaluated peripheral hemodynamic responses in 35 young male participants using a between-subjects design, where participants underwent partial and complete blood-flow occlusion, both absolute [50 mmHg (G1) and 250 mmHg (G3)] and individualized (systolic blood pressure + 50 mmHg, G2), in the left arm under resting conditions (Desanlis et al., 2024). The protocol applied 3 intervals of 7 min of pressure, separated by 10–20 min of rest, while assessing tissue oxygenation (TSI), oxyhemoglobin (O_2_Hb), and deoxyhemoglobin (HHb) using near-infrared spectroscopy (NIRS). Their findings demonstrated greater deoxygenation and faster reoxygenation in participants subjected to occlusion pressures exceeding systolic blood pressure (G2 and G3) compared to partial occlusion (G1), with no significant differences between the G2 and G3 groups. The authors concluded that individualizing pressure provides the optimal response to IPC and that 250 mmHg may be excessive.

The most significant issue we identified is that the between-subjects methodology and the lack of true personalization of applied pressures limit the ability to draw firm conclusions regarding the impact of IPC pressure on tissue oxygenation responses. Since the authors employed a between-subjects design, participants were randomized to one of three conditions rather than undergoing each experimental condition.

Although the authors aimed to personalize the applied pressure by adjusting it relative to systolic blood pressure, the absence of detailed reporting—such as each participant’s arm circumference and the cuff width used to determine systolic blood pressure—raises concerns about whether the pressures applied were truly individualized. Systolic blood pressure can only be equated to limb occlusion pressure when the cuff width and bladder type match those used to measure systolic blood pressure (Rolnick et al., 2021; Rolnick et al., 2023). For example, a narrower cuff would lead to higher-than-expected pressures, whereas a wider cuff would require less pressure to achieve occlusion (Graham et al., 1993).

Moreover, since the limb circumferences of the participants were not reported, we question whether the individualized pressure (G2) was accurately applied across subjects. The same pressure increase (e.g., 50 mmHg) could produce varying physiological effects depending on limb size (Jessee et al., 2016). This issue is especially relevant in a between-subjects design compared to a within-subjects design, where each participant would experience all conditions and act as their own control. Although arm circumference would still be useful to report in a within-subjects design, its importance diminishes as each participant’s response can be directly compared across conditions.

Last, it is important to note that the authors did not address the limitations of true personalization in their limitations section. Specifically, the inability to fully personalize pressure without reporting key device-related characteristics, such as cuff width and bladder design, means that their “individualized” pressure prescription was only likely partially reflective of the impact of the different pressure schemes. This oversight further reduces confidence in the study’s conclusions regarding the optimal approach to IPC pressure prescription.

In studies such as this, where the primary aim is to determine the effect of applied pressure on tissue oxygenation responses, a within-subjects design would better address individual differences in limb size, thereby enhancing the precision of NIRS data and the overall validity of the findings.

## Discussion

Given the influence of cuff characteristics on tissue pressure, it is crucial to report the specific characteristics of the device used in studies involving blood flow restriction or IPC training. This improves the interpretation of results, particularly when the independent variable of interest is pressure. In the study by Desanlis et al. (2024), it would have been beneficial to include participants’ arm circumference measurements, considering that the use of arbitrary pressures could result in variable responses due to differences in limb circumference.
